# Caregiver Experiences Navigating the Diagnostic Journey in a Rapidly Progressing Dementia

**DOI:** 10.1177/08919887221135552

**Published:** 2022-11-22

**Authors:** Alissa Bernstein Sideman, Joni Gilissen, Krista L Harrison, Sarah B Garrett, Michael J Terranova, Christine S Ritchie, Michael D Geschwind

**Affiliations:** 1 Institute for Health Policy Studies, University of California, San Francisco, CA, USA; 2 Global Brain Health Institute, University of California, San Francisco, CA, USA; 3 Department of Humanities and Social Sciences, 8785 University of California San Francisco, San Francisco, CA, USA; 4 Department Family Medicine & Chronic Care, 70493 Vrije Universiteit Brussel(VUB), Belgium; 5 Division of Geriatrics, University of California, San Francisco, CA, USA; 6 Department of Neurology, 8785 University of California San Francisco, San Francisco, CA, USA; 7 The Mongan Institute and the Division of Palliative Care and Geriatric Medicine, 2348 Massachusetts General Hospital, Boston, MA, USA

**Keywords:** dementia, rapidly progressing disease, prion, jakob-creutzfeldt, misdiagnosis, caregiver, diagnosis

## Abstract

**Introduction:**

People with suspected Alzheimer’s disease and related dementias (ADRD) and their families experience a burdensome process while seeking a diagnosis. These challenges are problematic in the most common dementia syndromes, but they can be even more distressing in rarer, atypical syndromes such as rapidly progressive dementias (RPDs), which can be fatal within months from onset. This study is an examination of the diagnostic journey experience from the perspective of caregivers of people who died from the prototypic RPD, sporadic Creutzfeldt-Jakob Disease (sCJD).

**Methods:**

eIn this mixed-methods study, qualitative data were drawn from interviews with former caregivers of 12 people who died from sCJD. Chart review data were drawn from research and clinical chart data about the person with sCJD. Data were analyzed by a multidisciplinary research team using qualitative and descriptive statistical analysis.

**Results:**

We identified 4 overarching themes that characterized the experience of the diagnostic journey in sCJD: clinician knowledge, clinician communication, experiences of uncertainty, and the caregiver as advocate. We also identified 4 phases along the diagnostic journey: recognition, the diagnostic workup, diagnosis, and post-diagnosis. Sub-themes within each phase include struggles to recognize what is wrong, complex processes of testing and referrals, delay and disclosure of diagnosis, and access to resources post-diagnosis.

**Conclusions:**

Findings suggest that more work is needed to improve clinician diagnostic knowledge and communication practices. Furthermore, caregivers need better support during the diagnostic journey. What we learn from studying sCJD and other RPDs is likely applicable to other more common dementias.

## Introduction

Underdiagnosis and misdiagnosis are common in Alzheimer’s disease and related dementias (ADRD).^1^ Often, people with suspected dementia and their families experience a burdensome process while seeking a diagnosis. This process may involve in-depth testing, multiple referrals, visits with numerous specialists, and incorrect diagnoses.^2,3^ In addition, most people are first seen by their primary care providers, who often do not have the time or resources to engage in a comprehensive diagnostic workup for dementia or may prioritize a patient’s other immediate needs.^4-13^ Those who are able to get a referral to neurology often have long wait times and experience many hurdles while seeking appropriate testing. Although these challenges are problematic in the most common dementia syndromes, such as Alzheimer’s disease, they can be even more distressing in rare and atypical dementia syndromes such as rapidly progressive dementias (RPDs) in which decline happens much more quickly.^14^

RPDs such as sporadic Creutzfeldt-Jakob disease (sCJD) are different than more common dementia syndromes in that they often can be fatal within weeks or months from onset, rather than the slower years-long progression in diseases such as Alzheimer’s.^15^ Sporadic CJD typically occurs in those who are aged 50-70 with a very short average time from symptom onset to death of 5 months, with 85% of patients dying within one year of symptom onset. Sporadic CJD involves an array of distressing symptoms that usually include cognitive decline, loss of motor control, behavioral changes (including sleep disturbances), visual disturbances, and many other features.^15^ Diagnosis can be difficult because depending on where in the brain the disease begins and spreads, patients can have a multitude of neurological and psychiatric symptoms. This is why some clinicians call sCJD “the great mimicker,” as it can appear similar to many other syndromes, particularly early on in the disease process.^16,17^ In one large study, patients with sCJD received an average of four misdiagnoses before being accurately diagnosed, and by the time they received a correct diagnosis they were as far as two-thirds of the way through the disease course.^16^ Delayed or missed diagnosis in a rare and rapidly progressing disease such as sCJD can lead to medical, physical, and psychological challenges for the patient and emotional and financial challenges for the family.^17-19^ An accurate diagnosis informs clinicians’ prognostic estimates and advice about what to expect, as well as the appropriate care and medications to take. Accurate diagnosis also enables patients and their families to make important decisions about the future and identify safety precautions in the home.

The purpose of this study was to use mixed methods to characterize the experiences of the diagnostic process in sCJD from the perspective of family caregivers of people who died from the disease, and to identify characteristics of patients with sCJD to contextualize their experiences. We also aimed to identify opportunities for better supporting people with sCJD throughout the process, which we refer to as the “diagnostic journey.”

## Methods

### Design and Setting

We conducted a qualitative and chart review study focused on the experiences and needs of twelve caregivers of people with sCJD recruited from the UCSF Memory and Aging Center (MAC) Rapidly Progressive Dementias (RPD) research program database. This manuscript specifically explores caregiver experiences of the diagnostic journey– from when a caregiver recognized there was something wrong, the diagnostic workup, and immediately post-diagnosis, though we recognize that the phases identified may overlap or occur iteratively. Research and clinical chart data about the person with sCJD further contextualizes the caregivers’ experiences.^20^ Qualitative data collection and reporting is consistent with the Consolidated Criteria for Reporting Qualitative Studies (COREQ) (Supplemental Materials).

### Participants and Recruitment

Participants for this study were recruited because they were caregivers for people with sCJD who were part of a cohort that had previously consented and enrolled in an RPD parent study approved by the University of California, San Francisco Institutional Review Board (IRB). The caregiver interview study was separately approved by the IRB. Participants in this current study also participated in a related study focusing on the palliative care needs in sCJD^21^ for which interview data was also used for this study. Details of the caregiver interviews, chart review, and data collection are also described in our sCJD palliative care study paper (20) but are summarized here as well. Caregivers were recruited via phone or email using purposive sampling from the UCSF Rapidly Progressive Dementia (RPD) Study database using a medical chart review process. Inclusion criteria included being a caregiver of someone who died from sCJD at least 3 months but no more than 3 years prior to the start of data collection and being conversational in English. We chose not to recruit individuals about whom research program staff or patient family members expressed concern regarding emotional or cognitive wellbeing. If a primary caregiver was not available (eg, because they were deceased or had cognitive impairment) we contacted the next listed caregiver for the patient. We reached out to 23 individuals; three declined to participate because they did not feel they had anything to add or did not want to revisit a painful time, and eight never replied to our emails or calls. We first recruited caregivers for interviews and then extracted chart data about the patient connected to that caregiver.

### Data Collection

#### Qualitative

An interdisciplinary team (social scientists, palliative care specialists, geriatrician, and behavioral neurologist) developed a qualitative interview guide focused on the following domains: (1) experiences along the disease trajectory; (2) caregiver activities and quality of life; (3) challenges and sources of distress; (4) sources of support; and (5) opportunities for improvement (Supplemental Materials). In-depth interviews were conducted via telephone between September 2019 and March 2020 by a sociologist (SBG) with expertise in qualitative data collection. Caregivers self-reported their demographic information. Analytic notes were taken during each interview and summarized into case summaries. Case summaries included a summary of the caregiver’s description of the person with sCJD’s disease course, the types of needs and challenges experienced, supports/resources desired, major themes, and fieldworker reflections on the conduct and quality of the interview.

#### Chart Review

For each caregiver in the study, two team members (JG, MT) extracted data from the connected patient’s UCSF Electronic Health Record, the Memory and Aging Center (MAC) research chart and records (which included outside records), and the UCSF RPD research database to identify: (1) patient demographics and disease characteristics; and (2) symptoms at time of first neurology encounter (cognition, function, neuropsychiatric symptoms and mood). The RPD research study chart included original research data forms, the RPD study patient visit note, cognitive and behavioral assessments, family history tree, CSF biomarkers, *PRNP* mutation and codon 129 analysis results, and for patients who had autopsy, sCJD molecular classification information.

### Data Analysis

Throughout the data collection process, the multidisciplinary team familiarized themselves with the data by meeting weekly to review case summaries and excerpts from transcripts to identify preliminary areas of focus. Based on this initial review, they created a codebook of deductive codes reflecting key concepts regarding challenges and sources of support and elements of the disease experience, such as diagnosis. Investigators (KLH, SBG, and CSR) iteratively refined the codes by double-coding transcripts and discussing discrepancies in coding until agreement was reached, then codes were applied to all transcripts (KLH).

For the current study, the following analyses were conducted and diverged from those in our palliative care study.^21^After “diagnosis” was identified as an area of focus across every interview conducted, two members of the team (ASB, JG) used both deductive and inductive thematic analysis using ATLAS.ti,^22^ a qualitative data analysis software, to analyze the transcripts specifically regarding this topic. They first coded all the data deductively for any excerpts in a transcript that related specifically to this focal area, tagging these excerpts with the deductive code “diagnostic journey.” These excerpts included discussions of experiences when caregivers recognized something was wrong, tried to identify a provider who could help make a diagnosis, experiences during the diagnostic workup and referral processes, experiences with diagnostic disclosure, as well as experiences immediately post-sCJD diagnosis. After reviewing the excerpts labeled “diagnostic journey,” and familiarizing themselves with the data surrounding this topic, they then independently inductively coded the transcripts to identify different aspects of the diagnostic journey and created a codebook based on these codes. They found agreement between how they characterized and defined the different stages of the diagnostic journey based on descriptions by caregivers in the study (eg, codes included such items as instigating event, testing and referral challenges, timing of diagnosis, diagnostic delivery). They used the codebook to define these different stages and the specific challenges and opportunities that emerged within these stages and iterated upon the codebook throughout the process. They then combined and organized codes to develop key themes within each stage of the diagnostic journey. They found four themes that did not fit neatly into any one category and instead spanned all categories: uncertainty, clinician knowledge, clinician communication, and caregiver as advocate. They then identified exemplary quotations for each of the themes and reviewed these quotations with the entire team. Changes were made based on insights from team members with specific expertise; for example, they teased out the distinction between the broader domain of “diagnostic delivery,” which refers to all aspects of giving and receiving a diagnosis, and “diagnostic disclosure” which was a theme that exemplified the experience of how the diagnosis was communicated by a healthcare provider. Once there was agreement about the stages and the themes, theme definitions, and examples, they created tables which were then reviewed by the team.

Chart-based data were abstracted, summarized quantitatively using descriptive statistics, and used to provide context to caregivers’ reports, including first symptoms, disease duration, and timing required to obtain a correct sCJD diagnosis.

## Results

### Participant Characteristics

Twelve caregivers were interviewed for this study. Caregiver and patient characteristics are reported in Table 1. Caregivers had a median age of 59 (range 45-73), half identified as female (50%) and as married or partnered (50%). Ten identified as White, two as Asian or other; and most caregivers had private insurance (75%) and a college- or post-graduate education (75%). Interviews occurred at a median of 22 months (range 11-39) after the person with sCJD died.

**Table 1.** Patient and Caregiver Characteristics.

Sociodemographic	Patients	Caregivers
	n = 12 (%)	n = 12 (%)
Age at data collection (mean [SD])	71.4 (8.8)	59 (45-73)
(Median [range])	70 (60-86)	—
Sex		
Female	7 (58)	6 (50)
Male	5 (42)	5 (42)
Race/Ethnicity^1^		
Asian	1 (8)	1 (8)
Latinx/Hispanic	1 (8)	0
White	10 (83)	9 (75)
Black/African American	0	0
Declined to report	0	1 (8)
Educational level		
Less than or equal to high school	5 (42)	0
High school to some college	3 (25)	2 (17)
College or graduate school	2 (17)	9 (75)
no record/declined	2 (17)	1 (8)
Marital status at time of data collection		
Married	9 (75)	6
Widowed	2 (17)	4
Divorced	0	1
Single	1 (8)	0
Income category (total household)		
$40,000 – <$60,000	—	2 (17)
$60,000 – <$80,000		1 (8)
$80,000 – <$100,000		1 (8)
$100,000+		6 (50)
Declined to report		2 (17)
Number of people in household (mean [SD]) (median [range])	—	2.5 (1.5)
		(1-6)
**Patient disease/health characteristics**		
Data from full UCSF RPD research study visit	8 (70%)	—
Data from inpatient records	4 (30%)	
Total disease duration from onset^2^ to death, months (mean [SD]) (median [range])	15.6 (11.7)	
	14.5 (4-41)	
Time between onset and diagnosis,^3^ months (mean [SD])	9.1 (7.8)	
(Median [range])	6.5 (1-25)	
Time between first UCSF visit to death, months (mean [SD]) (median [range]))	5.1 (7.4)	
	2.5 (0-26)	
Time from diagnosis to death, months (mean [SD]) (median [range]))	6.1 (7.9)	
	3.0 (0-27)	
**Predominant 1** ^**st**^ **symptom category reported in patient file†**	**# Of subjects**	—
Reduced ability to plan and solve problems	1	
Gait, imbalance, and falls	5	
Delusion, hallucinations, aggression	2	
Changes in voice, speaking, language	2	
Visual changes	3	
Forgetfulness	2	
**Patient cognitive and physical functioning and symptoms at time of first visit at UCSF**		
(n=8 who participated in a 2-day outpatient research visit**)**	—	
Mini-mental state examination (MMSE),^4^ (mean [SD])	13.5 (8.9)	—
(Median [range])	12.5 (0-25)	
Medical research Council (MRC) prion disease rating scale^5^ (mean [SD]) (median [range])	13 (5.4)	
	14 (3-19)	
Barthel index^6^ (mean [SD])	66.3 (36.2)	
(Median [range])	77.5 (0-100)	
Clinical dementia rating (CDR) scale^7^ composite sum of boxes score (mean [SD])	11.1 (5.2)	
(Median [range])	11 (3.5 -18)	
Neuropsychiatric inventory questionnaire (NPI-Q)^8^ composite score (mean [SD]) (median [range])	9.3 (6.8)	
	6 (4-24)	
Geriatric depression scale (GDS) long form^9^ (mean [SD])	8.2 (5.6)	
(Median [range])	9 (2-15)	
**Prion type and diagnosis (** **n** **=12)**	—	
PRNP gene pathogenic mutation Analysis^10^ showed no mutation, n	12 (100%)	—
Codon 129 type	—	
129 M/V	5 (42)	—
129 V/V	3 (25)	
129 M/M	4 (33)	
MRI diagnostic for CJD	11 (1 not diagnostic)	
CSF positive for sCJD on RT-QuIC test	11 (1 no LP)	
Positive sCJD diagnosis after autopsy tissue pathology testing	11 positive (1 refused)	

Acronyms: RPD rapidly progressive dementias; MAC, Memory and Aging Center; sCJD sporadic Creutzfeldt Jakob’s disease; LP, lumbar puncture; *PRNP*, prion protein gene.

Molecular classification of sCJD includes codon 129 polymorphism and the type of prion – 1, 2 or mixed 1-2 – as shown on Western blot after partial digestion of prions with proteinase K, and is based on the molecular weight and glycosylation pattern of the resultant prion fragment.

†More than 1 symptom can apply as some patients had more than 1 at onset.

Missing data: Patients: age at time of visit (n=1); educational level (n=1); MRC prion rating scale (n=1); CDR scale (n=1); NPI-Q (n=1); GDS scale (n=3 because scale could not be administered due to patient factors); MRI (n=1); RT-QuIC test (n=1); T-tau (n=1); 14-3-3 protein (n=1); autopsy test (n=1 because family refused). Caregivers: 1 who refused demographic survey.

^1^ Race/ethnicity data collected to report per funder requirements. Caregiver data was self-reported and categorized based on NIH reporting categories.

^2^ “Onset’ as identified by the treating neurologist in the patient file at the earliest symptom we could identify based on available medical records and the medical history obtained during our research visits.

^3^ ‘Diagnosis’ as identified by the treating neurologist in the patient file (could have been at UCSF or elsewhere), based on own assessment, reports from other physicians send to neurologist or highlighted by caregiver or patient during UCSF visit.

^4^ Mini-Mental State Examination (MMSE) total score range: 0-30, with higher scores indicting more normal cognition.

^5^ Medical Research Council (MRC) prion disease rating scale^4^.

total 0-20, with lower scores indicating worse function.

^6^ Barthel Intext total score range: 0-100, with lower scores resembling greater dependency.

^7^ Clinical Dementia Rating (CDR) scale composite score, total score range: 0-18, with higher scores indicating more impairment.

^8^ Neuropsychiatric Inventory Questionnaire (NPI-Q) composite score, total score range 0-36, with higher scores indicate more severe neuropsychiatric symptoms.

^9^ Geriatric Depression Scale (GDS) Long Form scale, total score range: 0-30, higher scores indicated screening positive for depression.

^10^ *PRNP* (prion protein) codon 129 genotypes of sCJD are MM: homozygous for methionine; MV: heterozygous for methionine and valine and VV: homozygous for valine.

Based on chart review, eight of the twelve patients came to UCSF specifically for participation in a two-day outpatient clinical research visit; the remaining four were admitted to the UCSF inpatient service (Table 1) and had limited contact with the UCSF RPD study team. Median age at first UCSF visit was 69 years old (range 60 – 86). Eleven of twelve had an MRI that was diagnostic for sCJD. The one patient with inadequate quality MRI was positive for 14-3-3, total Tau (>4000 pg/mL), CSF RT-QuIC test and was pathology-proven. Eleven of twelve had a positive CSF RT-QuIC (1 had no lumbar puncture), and eleven autopsied cases were ultimately pathology-proven. The one patient without autopsy had negative 14-3-3, and total Tau, but a positive MRI and CSF RT-QuIC, meeting UCSF, European 2009, and European 2017 probable sCJD criteria.^23^ Sporadic CJD molecular subtypes included one patient with MM (not pathology proven), three with MM1, three with MV1-2, two with MV2, one with VV1-2, and two with VV2. The median overall disease duration was 14.5 months (range 4-41). The median time between UCSF visit and death was 2.5 months (range 0-26); half of those died within 2 months.

### Issues That Occurred at all Phases of the Diagnostic Journey

We identified four ovearching themes that were present at every stage of the diagnostic journey: experiences of uncertainty, clinician knowledge, clinician communication, and caregiver as advocate. We provide additional representative examples in Table 2.

**Table 2.** The Diagnostic Journey in sCJD.

**Overarching themes**	**Exemplary quotations**
Experiences of uncertainty	CG03: God. It was really awful. It was stressful. First of all we didn’t know what it was, and then it was the question of is this real or is it psychological…at 1 point and I was just frustrated because it was kind of like between what the doctors were telling me and [husband, patient] and what we were seeing it was so different.
Clinician knowledge	**Lack of knowledge-** CG07: They said, “no, there’s nothing,” they just started kind of taking blood pressure and doing this stuff and he was kind of fine answering questions and they let him go and I wasn’t happy with it and I argued with the staff and I said, "Something’s wrong.” and they said, “sorry, we can’t help you.”
	**Knowledgeable-** CG02: I mean, I felt so secure with those people. They knew everything. They knew what they were doing…We went from doctors who didn’t know anything to doctors who-- they knew everything and how to deal with it. They knew all the answers, and I was like “wow.” that was comforting
Clinician communication	CG05: If somebody didn’t have experience or a medical background, I don’t know if they would have gotten there as quickly and I think he probably was going there…I think it needs to be very clear. You can say, “I can’t cure this, there are no meds.” well I understand that to mean, “You’re going to die and you’re dying soon.”
Caregiver as advocate	CG04: I Interviewed doctors. I Interviewed researchers. I Interviewed people-- I did cold calling…I mean, I was phone banking from 6 in the morning till twelve midnight trying to get a person on the phone
**Diagnostic journey stage**	
Recognition phase	
Struggling to identify what is wrong	CG03: At times he was zoned out a little, but I kept on thinking he was still playing tennis, walking, driving, all those things
Sentinel events	CG04: We kept on managing…and then a major event happened where he went to the bank… he had gone to the bank and withdrawn all the money…saying that the bank was having people steal and he wanted to make sure that all his money was still there.
Gatekeeper clinicians	CG03: I atill harbor anger with [health center1]…the fact that this neurologist said it was psychological…and asking over and over and over again for a year to be sent to [specialty clinic] to figure out what’s really going on here with him, and they wouldn’t do it… it wouldn’t have changed the diagnosis, but it would have changed his end of life in terms of how we would have had conversations and knowing where we were headed
Diagnostic workup	
Clarifying early symptoms	CG01: I had noticed the last year or so I was up there [patient] was sometimes forgetting things. I Thought it was just because she was living under so much stress and anxiety that maybe she just was not listening to me good and therefore was not retaining information.
Challenges with testing and referrals	CG07: I feel like I twisted this guy’s arm to write a recommendation to be seen past the initial testing and he did it, he did it because of the argument that we had and it was something to the fact of, “I’m actually not leaving here unless you do what’s right because if this was your dad or if this was your grandfather, you would want to see more care and you’re just passing me through the system and I can’t have that happen
	CG02: And that was our whole thing that so going from doctor to doctor and from hospital to hospital we couldn’t believe that there wasn’t just 1 form that answered all these questions that me and my sister could have all filled out the form and remembered through the years what symptoms my dad had
Diagnostic delivery	
Diagnostic disclosure	CG05: I still go back to if somebody is 99.9% diagnosed with CJD, no matter the type, when they leave that [specialty clinic] clinic they need a piece of paper that’s very plain that goes A, B, C, D. I told you this, now I’m telling it to you again because what they hear and what they read are 2 different things
Timing of diagnosis	C03: Earlier diagnosis would have happened at the time when we could have had deeper conversations. By the time we know what was really going on he was at a point he could barely communicate.”
Patient and family emotional reactions to disease	**Disbelief:** CG11: I was completely in disbelief, and I knew nothing of CJD. Needless to say, I said, “well, we’re going to go get a second opinion."
	**Fears:** CG10: The other damning thing about this disease is to know the variant that she had, because if she had the genetic variant, then family members were at risk…which, again, was very difficult to deal with
	**Resolution:** CG12: He paused for a moment, and he said “well, based on what we can see I think you have another 6 to 9 months"-- he said “6 to 12 months with your wife,” which was a relief. She was gonna be off the keppra, and I had 6 to 12 months with her. It turned out she passed right around 9 months later, but I at least felt like I can relax. I Can focus on her and making sure that-- I don’t have to be on top of her
Post-diagnosis	
Access to resources, education, and anticipatory guidance	C11: “We still went through the entire 2 days to learn about the disease. That, to me, was more important. To learn about it. How do I deal with this?”
Caregiver retrospective	CG12: “When you look and you look back, I feel that this was going on a lot longer than either 1 of us realized.”

#### Experiences of Uncertainty

Most caregivers reported experiencing uncertainty across every stage of the diagnostic journey: when trying to figure out what was wrong, finding the right tests, and understanding the disease. They also noted that non-specialist clinicians were often uncertain of the diagnosis through much of the disease process.

CG3: God. It was really awful. It was stressful. First of all we didn't know what it was, and then it was the question of is this real or is it psychological… I was just frustrated because it was kind of like between what the doctors were telling [us] and what we were seeing it was so different.

#### Clinician Knowledge

Most caregivers reported that they struggled with clinicians who were not knowledgable about the possible causes of symptoms.

CG2: I thought I was going to have an aneurysm…I mean, you want to scream…These doctors have no idea, not even a nurse, saying "You know, I saw somebody that had these similar signs, and they found out that it was CJD." Nothing. There's staff doctors coming every day. There's five of them, and they have no guess?

When caregivers did encounter clinicians with prior familiarity with prion disease, or were finally able to see a specialist, they felt that these clinicians were especially important in their experience, and served as pivotal people in the diagnostic journey. Knowledgable clinicians helped to diminish the uncertainty felt.

#### Clinician Communication

Issues around clinician communication were identified at many stages of the diagnostic journey. When caregivers felt that clinicians failed at communication, they specifically noted clinicians not taking time to communicate clearly or felt a lack of empathy.

CG 10: She was upset because I think she felt like [the doctor] was just so frank…I still to this day don't know what she was expecting for him to say or maybe she felt like he could have been a little more compassionate.

When clinicians communicated well, caregivers felt they gave clear feedback on what was happening, responded to emails or phone calls quickly, or expressed appropriate gravity over the diagnosis.

#### Caregiver as Advocate

Nearly all of the caregivers in our sample chose to become engaged advocates for the patient and drove the processes involved in the diagnostic journey. In the early stages, this took the form of advocating to a clinician that something was wrong, advocating for more tests, or gaining an education about potential causes.

CG06: It was pretty much me who was on top of all of it, I got involved and started taking care of all that was going on – all the research and stuff.

Caregivers also reported having to do their own research to try to identify resources, support, and information.

CG07: I started to do my own research and started looking online and trying to be-- I felt like I didn't have information and so I was trying to arm myself and I'm not a doctor, so I really wanted to know so much about it to where I actually knew how to ask good questions.

Most caregivers we spoke with had to navigate the complex terrain of figuring out what was wrong, being an advocate for the patient, and moving through the diagnostic process.

### Four Phases of the Diagnostic Journey

We identified four phases in the diagnostic journey that were most salient based on caregiver experiences: (1) the recognition phase; (2) the diagnostic workup; (3) diagnostic delivery; (4) and post-diagnosis. Each phase had sub-themes (Table 2).

#### Recognition

##### Struggling to Identify What Is Wrong

All participants reported going through an early struggle to identify what was wrong with the patient, as well as uncertainty determining the meaning of early symptoms and disentangling symptoms from other co-existing circumstances, as noted under the theme focused on experiences of uncertainty above. Co-existing circumstances could be mental health challenges, stressful social or work-related situations, or other challenges in the patient’s life that seemed to overlap with worsening symptoms. The uncertainty during this early phase was a source of distress for caregivers.

##### Sentinel Events

Caregivers identified two major sources of impetus for action as they struggled to identify what was wrong. First, a sentinel event that was a marked difference from how things were going previously, for example, a neighbor calling out erratic behavior, financial mismanagement, or a car accident.

CG11: I think the wakeup call for her was when they were driving together…he got on the ramp on a road on a major highway actually in the opposite direction.”

Sentinel events noted in patients’ charts via chart review included: stroke, wanting to withdraw large sums of money, trouble navigating, driving incidents, not being able to find the bathroom in one’s own house, and not recognizing one’s own health limitations. In many cases, sentinel events led caregivers to spend more time with the patient, enabling them to notice changes and seek care. Each caregiver identified a specific moment when they became certain that something was wrong.

##### Gatekeeper Clinicians

Among patients who brought their concerns to their primary care provider (PCP), most reported the clinician either dismissed their concerns or was perplexed by the symptoms. These clinicians were often the gatekeepers to next steps in the diagnostic workup. A caregiver explained this dilemma, reflecting on both her own, and the general practitioner’s, lack of clarity.

CG1: My sister [patient] had a very, very hard life and so I chalked a lot up to her behavior as being to a lot of stress and depression that she was experiencing the last couple of years of her life even before this was diagnosed. But who would have known, there was just no way I could have ever have suspected that. And she was seeing a general practitioner who kept saying there was nothing wrong with her.

Most gatekeeper clinicians did not know what to do and caregivers felt distressed by the experience trying to figure out what was wrong. There were exceptions, however, in which some PCPs took initiative to do a full workup and reacted with urgency, particularly after viewing MRI results.

#### Diagnostic Workup

##### Clarifying early symptoms

Part of diagnostic journey in sCJD is identifying and clarifying when symptoms began and what they were, in order to determine the type of disease and to help ascertain prognosis. Chart review data indicated most common first symptoms were forgetfulness or slowness of thinking (Table 1), which is consistent with the larger UCSF sCJD cohort in which 20% of patients have cognitive dysfunction as their first symptoms.^24^

##### Challenges with Testing And Referrals

In interviews, caregivers described the challenges finding someone with sufficient expertise to suspect prion disease. Caregivers highlighted sources of stress that included: waiting for tests and test results; lack of communication about what tests were for and meant; difficulty or delays with referrals, and challenges with transportation. Some caregivers felt clinicians lacked the appropriate urgency to figure out a diagnosis given how quickly the patient was deteriorating. Most participants received many misdiagnoses along the way. Only a few caregivers described simple journeys to an accurate diagnosis.

CG2: [The doctor] was going to get back to us, but then he never did, and it was kind of bad. That part was really bad…at times I thought "Oh my God, I'm going to call 911 and I'm going to get an ambulance to come to [health center 1] and take my dad to [health center 2]'s ER so that they will take an MRI and have their radiologists look at it and tell us what's wrong, because there's something going on.

Chart review data indicated a wide range of elapsed time – ranging from 1-25 months (median 6, IQR 9) between date of first symptom to correct diagnosis.

#### Diagnostic Delivery

##### Diagnostic Disclosure

Some caregivers reported frustration with how diagnosis and/or confirmation of diagnosis were disclosed, including those who reported a feeling of clinician insensitivity given the rapid and devastating nature of the diagnosis. Several caregivers wanted, but did not receive, estimates about prognosis. Some wanted information to be presented directly,

CG5: I think it would be very helpful [to have it stated] in true layman's terms, very simplistic... “You have CJD, no known cause, no treatment, no outcome short of death.”

Others, however, were unsure about what approach would have been most helpful. Even for caregivers who were not entirely sure what approach to diagnostic disclosure would have eased the experience, there was still some sense of unease about what transpired during the disclosure process.

##### Timing of Diagnosis

Chart review indicated patients were first evaluated by the UCSF RPD study (when they either received their diagnosis or had it confirmed) at a wide range of disease severity or degree of functional impairment. The median time to diagnosis was nine months between disease onset and diagnosis (range 1-25). Of the twelve total patients, eight were seen for full RPD research visits in which more detailed and prion-specific data was collected, including the MMSE and the Barthel Index. On the MMSE, three were mildly impaired (21-25), two were moderately impaired (10-20), and three were severely impaired (0-10), and on the Barthel Index, four were independent (80-100), one was minimally (60-79) and two were partially (40-59) dependent, and one was totally dependent (0-19). The four patients on the inpatient service, and who did not have full RPD assessments including Barthel and cognitive testing, were all severely impaired. Overall, the full disease trajectory ranged from 3 to 41 months (1.2 years), with a median of 15.5 months.

Caregivers reported distress about the length of time taken to receive a diagnosis. Many felt that diagnosis occurred too late to permit informed conversations about care plans and end of life preferences.

CG3: Earlier diagnosis would have happened at the time when we could have had deeper conversations. By the time we knew what was really going on, he was at a point he could barely communicate.

Both interviews and chart data indicated most patients were close to death by diagnosis, which is consistent with our prior data showing patients with sCJD are on average two-thirds of the way through their disease course by the time the diagnosis of sCJD becomes their leading diagnosis.^25^

##### Patient and Family Emotional Reactions To Disease

Many patients and caregivers responded to diagnostic disclosure with denial, disbelief, or fear. However, one caregiver reported that the patient responded surprisingly matter-of-factly given the implicatons of the diagnosis, and this curtailed the caregiver’s own response. When patients or family members responded to diagnosis with denial, it inhibited sharing information with friends or pursuing other tests or treatments, and was problematic when there were different responses among family members.

CG10: I don’t think my daughter accepted what her mother had. She always questioned it, questioned me. It was really a difficult time with her because she didn’t want to accept that her mom was-- had a fatal-- terminal illness.

Some caregivers felt such disbelief that they sought additional opinions. Others reported that the diagnosis opened fears about the possibility of other family members being genetic carriers of the disease and were distressed during the long wait-time for the post-mortem tissue analysis to provide a final diagnosis of sCJD.

Diagnosis, however, also had major benefits for patients and families, particularly in providing resolution to the uncertainty of what was wrong. This often happened when they reached a specialty center that had the ability to provide an accurate diagnosis.

CG2: We all felt very blessed and comfortable and at ease now that he was in the right place with the right doctors and that what we wanted to accomplish will start getting done.

Many expressed that the diagnosis ultimately provided a resolution to a mystery and validation of their concerns.

#### Post-Diagnosis

##### Access to resources, education, and anticipatory guidance

For all caregivers, once a diagnosis was made they felt they were finally able to gain access to resources, education, and information essential for care decisions, including enrolling in hospice care.

CG3: I got very good help post-diagnosis, but before that I just didn't know whom to ask.

The post-diagnosis phase enabled contact with specialists in RPD for advice about the disease, care, and medications. Post-diagnosis, caregivers also could access support commuities, such as the CJD Foundation (www.cjdfoundation.org), online support groups, and other caregivers of people with CJD.

##### Caregiver Retrospective

Once the diagnosis was known, some caregivers regretted the time spent obtaining tests and treatments that, in retrospect, seemed burdensome or inappropriate.

CG3: I still harbor anger with [health center1], the fact that they wouldn't send us [to a specialist], the fact that this neurologist said it was psychological, I mean all those kinds of things, and asking over and over and over again for a year to be sent to [specialty medical center] to figure out what’s really going on...I mean it wouldn't have changed the diagnosis, but it would have changed his end-of-life in terms of how we would have had conversations and knowing where we were headed.

Interview data also showed that many caregivers wondered if there had been earlier signs that they missed or did not act upon. They reflected on other circumstances such as stress, business changes, or another illness as reason they did not identify problems earlier.

CG1: Well, I guess hindsight's, you know-- I wished I would have known when my sister was forgetting things that we could have been looking at a problem but never did it cross my mind, never did it cross my mind that we were dealing with some kind of a disease.

## Discussion

This study reveals the challenges of the sCJD diagnostic journey. We identified overarching themes at all stages of the diagnostic journey and found that caregivers discussed the diagnostic journey in four major phases. We found that a diagnosis helped solve a mystery for patients and families, enabling a focus on symptom management and being together rather than figuring out or trying to reverse the problem. Furthermore, diagnosis provided an entry-point for expert clinical advice, including prognosis and anticipatory guidance, identifying correct medications to manage symptoms, and referrals to appropriate care models, such as hospice. Finally, diagnosis was an entry point into a community of caregivers as well as access to relevant resources, such as obtaining access to support groups, information about the disease through national disease-specific support organizations. By assessing caregiver experiences and the associated patient data, we identify opportunities for better supporting people with sCJD throughout the diagnostic journey, including building clinician capacity in rare diseases, improving the communication between primary care providers and specialists when addressing the diagnosis and care for rare diseases, especially those with short prognoses, and streamlining referral and testing procedures. This information will likely be useful for managing more common neurodegenerative diseases, as well.

Past literature has explored the challenges of the diagnostic journey in dementia more broadly, echoing the themes discussed here, such as the experience of the assessment process, diagnostic disclosure, and the changes in care that resulted from receiving a diagnosis.^26^ Unlike the conditions studied previously, sCJD is a rapidly progressing and rare disease, and caregivers reported distress around how little time they had left once the diagnosis was identified, expressing regrets about the complex testing and referral processes. We identify two key areas of focus and actions that could be taken to augment clinician practice in rare diseases such as sCJD and improve the experiences of patients and caregivers during the diagnostic journey.

## Clinician Knowledge and Communication

Clinician knowledge and communication are key targets for improving the diagnostic journey for patients with rare and rapidly progressing diseases. Although clinicians encounter many illnesses of unknown cause, there may be justification for increasing clinician familiarity with rare diseases, such as sCJD, even in the absence of treatments. That half of the patients in this study died within 2 months of being seen at UCSF suggests a lengthy time from disease onset to referral to a prion expert. Even the recognition that an illness does not fit any common disease categories could be sufficient to justify a more streamlined testing and referral process for patients with unknown ailments that appear to be rapidly progressing. Primary care providers and community neurologists may also need more guidance surrounding the diagnostic workup in atypical dementias that could lead to more rapid identification of a rapidly progressive dementia and its etiology. This workup may involve clarifying first symptoms, both their nature and timing, as well as whether the progression from onset is gradual or rapid, doing cognitive testing, imaging, and labs, and obtaining an extensive patient history. Clinicians may also need additional education in symptom presentation and prognosis in different dementias.^16,17,27^

Although it may be challenging for clinicians to arrive at a definitive diagnosis of a rapidly progressive dementia more quickly given how rare they are, there is a clear need to improve communication when dealing with diseases with uncertain origin, particularly prior to identifying the diagnosis. This improvement in communication is needed both between clinicians (for example, between primary care providers and neurologists during referral and diagnostic processes)**,** as well as between clinicians and patients and their families. Non-specialist clinicians may be just as uncertain as families are and could benefit from consults with specialists or participation in didactics and case review through programs such as The Alzheimer’s and Dementia Care ECHO Program for Clinicians (https://www.alz.org/professionals/health-systems-clinicians/echo-alzheimers-dementia-care-program) in which they can have more exposure to neurology experts and become more familiar with atypical dementias. Clinicians could also benefit from the use of serious illness guidance, which is a powerful tool for assisting in delivering information about uncertainty, as well as a diagnosis, no matter how preliminary.^28^ We suggest that clinicians should be ready and able to provide both emotional support and recommendations for where to identify reliable information, support services, and next steps. Taking best practices from knowledgeable clinicians who work with rare, rapidly progressing, or serious and incurable illnesses can provide insights into communication best-practices that can be implemented more broadly. Finally, clinicians (both neurologists and non-specialists) may benefit from more training in basic palliative care in order to work with families as early as possible in the disease progression to provide anticipatory guidance, education, and advance care planning and goal setting.^21,29^

## Supporting the Caregiver as Advocate

In our study, we found that caregivers of people with sCJD took on advocacy roles during the diagnostic journey. They did in-depth research, identified first symptoms, and dealt with a huge amount of burden. In prior studies of atypical dementias, caregivers of people with sCJD and behavioral variant frontal temporal dementia (bvFTD) were found to have the highest caregiver burden scores, measured using the Zarit Burden Inventory, as compared to those with Alzheimer’s disease.^30^ There is a need for better caregiver support while experiencing diagnostic uncertainty and in the post-diagnosis phase, as well as after a patient's death to address caregiver regrets, concerns, and challenges. Clinicians, both specialists and those in primary care settings, could help caregivers connect to support groups or psychologists with expertise in the area, connect to palliative care teams post-diagnosis, provide opportunities to receive additional counseling or support from social workers, and connect caregivers to care navigation programs such as the Care Ecosystem.^31^

## Limitations

Our study had several limitations. Recruitment ended prematurely due to the start of the COVID-19 pandemic, and we therefore enrolled a small sample. Three of the 23 individuals contacted for the study declined, and eight of the 23 individuals could not be contacted, which could limit conclusions as we do not know how similar they are from the 12 caregivers who agreed to participate. Our sample may not be representative, as molecular subtypes of sCJD do not match current international prevalence (eg, low MM1 recruitment), and furthermore, people who participate in extensive research studies, such as our 2-day outpatient RPD Study (although one-third of our cohort were from patients transferred or admitted directly to our inpatient service), are particularly well-resourced as demonstrated in our demographic characteristics, and therefore are likely not representative of all people with sCJD. Furthermore, none of the twelve participants were from underrepresented populations. Given the lack of diversity in our sample, challenges related to resources, structural racism, healthcare access, education, or health literacy are not adequately represented. Future work should explore the experience in rapidly progressive dementias in a larger and more diverse sample.

## Conclusion

Our findings suggest that clinicians need more education regarding how to recognize and streamline the testing and referral processes in rapidly progressing serious illnesses. Specifically, increasing awareness of rare diseases such as sCJD among primary care providers may help with faster referrals and lead to a more rapid diagnosis. Furthermore, a compassionate diagnostic process is needed in prion disease, with clinician communication informed by serious illness guidance.^32^ A streamlined diagnostic process may have positive impact on the patient and caregiver experience, even in the absence of treatment and cures, but particularly in the era of treatment studies.

## Supplemental Material

Supplemental Material - Caregiver Experiences Navigating the Diagnostic Journey in a Rapidly Progressing Dementia Supplemental Material for Caregiver Experiences Navigating the Diagnostic Journey in a Rapidly Progressing Dementia by Alissa Bernstein Sideman, Joni Gilissen, Krista L Harrison, Sarah B Garrett, Michael J Terranova, Christine S Ritchie, and Michael D Geschwind in Journal of Geriatric Psychiatry and Neurology
