# Supplementary material for: Caregiver Experiences Navigating the Diagnostic Journey in a Rapidly Progressing Dementia
Source: J Geriatr Psychiatry Neurol. 2022 Nov 22;36(4):282–94. doi: 10.1177/08919887221135552 (PMC10265278; doi:10.1177/08919887221135552)
Supplement: Supplemental Material - Caregiver Experiences Navigating the Diagnostic Journey in a Rapidly Progressing Dementia [file sj-pdf-1-jgp-10.1177_08919887221135552.pdf]

Supplemental table 1: Consolidated criteria for reporting qualitative studies (COREQ): 32-item checklist

No	Item	Guide questions/description
**Domain 1: Research team and reflexivity**		
Personal Characteristics		
1.	Interviewer/facilitator	PhD-level social scientist
2.	Credentials	PhD
3.	Occupation	Research sociologist
4.	Gender	Woman
5.	Experience and training	Doctoral-level training in sociology. Intensive expertise with collecting and analyzing in-depth interview data.
Relationship with participants		
6.	Relationship established	The relationship commenced with study recruitment and lasted for the duration of the interview.
7.	Participant knowledge of the interviewer	Participants understood the interviewer's and study goals: To learn about needs and experiences of persons with sporadic Creutzfeldt-Jakob disease (sCJD) & their families, hopefully to inform new or enhanced services at the specialty dementia clinic center.
8.	Interviewer characteristics	Interviewers identified and reported no personal characteristics that would contribute to bias or interests beyond their interest to contribute to scientific knowledge.
**Domain 2: study design**		
Theoretical framework		
9.	Methodological orientation and theory	The study draws on implementation science, with respect to trying to understand potential-user needs and wishes in order to inform future interventions.<br><br>The project also is based in subtle/critical realism, acknowledging both subjectivity in describing reality, the socially-constructed nature of reality; and also the existence of a reality that can be

		measured through the thoughtful collection of data and triangulation in analysis.<br><br>Study design was also influenced by the interprofessional, international study team, which included people with training in public health, health policy, ethics, sociology, anthropology, behavioral neurology, neuroscience, geriatrics, hospice and palliative medicine.
Participant selection		
10.	Sampling	Purposive sampling in order to interview individuals who cared for individuals with various genetic variants of sCJD who had died at least 3 months before data collection. Potential participants were identified based on data captured on patients with sCJD in the University of California San Francisco (UCSF) Rapidly Progressive Dementia (RPD) Study Database. Caregiver participants were recruited from among those who were listed as contacts for patients in the CJD database. We aimed to capture variation in degree of interaction with the UCSF RPD research team (e.g. patient who was participant in 2-day research study vs. inpatient clinical care) and to capture variation in clinical presentation through sCJD molecular classification. Inclusion criteria included being conversational in English and identifying as someone who provided care to the person with sCJD. Exclusion criteria included the patient lacking a caregiver candidate or candidate cognitive impairment. In cases where the primary caregiver was unavailable (e.g., due to death or cognitive impairment) we contacted the next available caregiver listed for the patient.
11.	Method of approach	The interviewer called or (in cases with no number or no working phone number) emailed candidates to share information about the study's purpose, design and procedures. The interviewer contacted the candidates up to three times in order to make contact.
12.	Sample size	12 bereaved caregivers for interviews
13.	Non-participation	23 individuals were contacted<br>• 3 declined, stating that they did not feel they had anything to add, or that they wished not to think about that period of their lives.<br>• 8 we emailed or left messages with but were unable to make contact with
Setting		

14.	Setting of data collection	Phone-based.
15.	Presence of non-participants	Non-participants were involved in 0 of the interviews.
16.	Description of sample	Interviews were conducted September 2019 to March 2020
Data collection		
17.	Interview guide	Interviewers had semi-structured interview guides that had been developed and pilot-tested in a related project. [[See below]]
18.	Repeat interviews	There were no repeat interviews.
19.	Audio/visual recording	With respondent permission we digitally recorded all interviews and had them professionally transcribed.
20.	Field notes	Field notes were taken during the interviews and included among study data. Interviewers also took notes following the conclusion of interviews as early summaries and analyses of the data.
21.	Duration	Interviews lasted a median of 88 minutes (range 41 minutes to 2h 41 minutes) or a mean of 92 minutes (standard deviation 31.5) each.
22.	Data saturation	The research team discussed data saturation at multiple points throughout the project.<br><br>The overarching information for which we sought to reach saturation was understanding domains of needs among patients and caregivers. We feel we have reached saturation on major domains.<br><br>We did not seek to reach data saturation with diagnostic journey lived experience (not possible) or domains by type of CJD (not possible with number per type).
23.	Transcripts returned	We did not return transcripts to participants.
**Domain 3: analysis and findings**		
Data analysis		
24.	Number of data coders	5 – KLH, SBG, CSR, JG, ABS

25.	Description of the coding tree	Primary deductive codes within the parent study were challenges, empirical helps, hypothetical helps, and prion-specific issues. We also coded for diagnostic journey, diagnostic disclosure, caregiving, and end of life/after.<br><br>Inductive codes within "diagnostic journey" included overarching themes (experience of uncertainty, clinician knowledge, clinician communication, and caregiver as advocate) and diagnostic journey stages (recognition, diagnostic workup, diagnostic delivery, and post-diagnosis)<br><br>Tertiary codes match the subthemes presented in the tables.
26.	Derivation of themes	An interdisciplinary, international team iteratively reviewed chart data, case summaries, and transcript excerpts throughout data collection to refine analytic approaches and identify preliminary themes. We conducted both deductive and inductive coding and analysis.<br><br>Overall themes were deductively identified based on domains of the interview guide (challenges, empirical helps, hypothetical helps). The code "diagnostic journey" emerged inductively, and the data was re-coded to identify inuctive codes within the diagnostic journey experience.<br><br>Categories and subthemes about the diagnostic journey were inductively identified based on iterative coding and discussion.
27.	Software	ATLAS.ti version 8
28.	Participant checking	No
Reporting		
29.	Quotations presented	Participant quotations are presented to illustrate themes / findings in the manuscript text and associated tables; each quotation is identified with a participant number
30.	Data and findings consistent	In our report we endeavor to maintain consistency between the data presented and the findings
31.	Clarity of major themes	We aimed to clearly present major themes in the findings
32.	Clarity of minor themes	We describe diverse cases and discuss minor themes in the text and table

Supplement 2: Interview framework

I want to remind you that you can stop the interview at any time. You can also refuse to answer any questions. Again, we will audio-record this interview in order to transcribe it for analysis. Are you willing to proceed with the interview and recording?

1. **Information about the Caregiver & Patient**

Objective: Explore the patient's disease trajectory; CG's relationship to and the support they provided to patient; explore other people who help the patient and/or CG.

- First, please tell me how you were related to [____name of patient]?
- Could you tell me when you first started noticing something changing with [patient]?
- Please give me a **brief overview** of what happened between then and when [patient] died.
  - Probe: What other providers or clinics during this period?
  - Who was the captain or quarterback of the patient's care during this period?

Now I'd like to ask you about your experience as a caregiver.

- What kinds of things did you do with or for [patient] in your day-to-day lives? *[if not clear from overview]*
- Did anyone else help you or your loved one?
  - Who helped?
  - How did they help?
- How would you describe your quality of life at that time? (Social, emotional, physical)
- Did you have any prior experience as caregiver for someone at EOL or with dementia?

2. **Knowledge, disease course & experiences**

Objective: Explore the degree to which they knew about the disease, the prognosis; how the disease would unfold.

Please think back to your experience receiving care.

- How did you learn the diagnosis?
- What did provider[s] communicate to you about the disease?
  - Did you receive guidance about what to expect over time?
  - Where did you get this information from? Do you wish it had happened differently?
- What aspects of your and the patient's lives in this period were difficult or distressing?

  Probe: Thank you for those answers. I'm going to share you a list of aspects of life with which caregivers sometimes have difficulty. *[[Discussion topic handout]]*
    - *Physical: The body, abilities, symptoms*
    - *Psychological: The mind, emotions, feelings*
    - *Social: Relationships, events, community*
    - *Cultural: Community or family traditions, values, preferences*
    - *Ethical: Fairness, morals, dilemmas about what is the "right" thing to do*
    - *Memory care services: Timing, location, services offered*
    - *Behavioral: How they engage with other people or their environment*
    - *Spiritual/religious: Meaning, purpose, relationship with God or higher power*
    - *Legal: Power of attorney, will, "getting affairs in order"*
    - *Financial: Power of attorney, will, "getting affairs in order"*
    - *Preparing for end-of-life: Location; types of services (e.g., hospice); loss or grief*

  - Did any of these feel difficult or distressing to you? *[Map re timing to EOL.]*
  - How did you handle these needs?

        - What helped you?
        - Where did [patient] receive services/support?
        - Who else was involved in these activities? (e.g. family, clinicians)
    - What could have made these difficulties easier or better for you/[patient]?

3. End of Life Experience

Objective: Understand CG's experience of patient's end of life period; their needs; whether the needs were met; how/whether they prepared for EOL.

- Did [patient] have any end-of-life needs that we haven't discussed? What were they?
- What needs did you have during this period?
    - [Probes: see list above]
- Are there types of services or supports that you wish were available to you or your family during this time?
- Did you and [patient] have an opportunity to discuss their goals for their care? Advanced care planning? Values or preferences for care at the end of life?
    - When did these start?
    - What led you to start these conversations when you did?
        - Do you feel that timing was too early, too late, or just right?
    - Were any clinicians involved in these discussions or preparations? How so? Who?
        - How did you feel about their being involved in this? OR
        - Would you have wanted them to be involved in this? How?

- Were there other things we haven't discussed that you had to or wanted to take care of before [____] passed? Please describe.
    - Were any clinicians involved in these activities/preparations? How so?
        - How did you feel about their being involved in this? OR
        - Would you have wanted them to be involved in this? How?
- Thinking back, do you wish any of these preparations or activities had gone differently? How? What would have made it easier or better?

4. Engagement with Palliative Care

Objective: Explore to what extent CG/patient engaged w/PC, how, why, and how that shaped the end of life period.

- Did you engage with anyone from services such as palliative care, symptom mgmt or hospice during this time?
    - *Offer to define palliative care.*
    - If yes: Please tell me about it.
        - Probes: What services provided? Why did you choose to engage them?
        - Would you make the same decision to engage /not engage again? Why/why not?
    - If no: Did you consider engaging with palliative care or related services at any point? Why/why not?
        - Would you make the same decision to not engage PC/related services again? Why/why not?

5. Caregiver as Expert

In this final section, we want to get your opinions as an expert. We're trying to build something to reduce suffering for people like you and [patient].

- What information do you wish you had known when [patient] was first diagnosed? OR
- What advice or information would you give another caregiver starting out on this journey?

Do you have any questions for me? Is there anything else I should have asked?

Please complete the following questions for study classification purposes.

1. What **year** were you born? _____________
2. Please mark one: □ Male □ Female □ Other
3. What is your current marital status?

□ Married/partnered
□ Not married, but living together
□ Widowed
□ Divorced or separated
□ Never married

4. How many people live in your household, including yourself? ________ *# of people*

5. Are you Latino or of Hispanic origin or descent?

□ Yes □ No

6. What is your racial background?

Please check **all** that apply:

□ White
□ Black/African-American
□ Asian
□ Native Hawaiian or other Pacific Islander
□ American Indian or Alaska Native
□ Other (please specify): _________________________

7. What is the highest level of school that you have completed, or the highest degree that you have received?

□ Less than high school
□ High school graduate
□ Some college/no degree
□ Associate degree/trade/vocational school
□ College graduate (Bachelor's degree)
□ Masters/PhD/professional degree

8. Are you a veteran?
   - □ No
   - □ Yes

9. Do you have health insurance?
   - □ No
   - □ Yes
   - □ I don’t know

10. If yes, what type of health insurance do you have?
   - □ Medicare
   - □ Medicaid/MediCal
   - □ Private
   - □ Other

11. How hard is it for you to pay for the very basics like food, housing, medical care, and heating? Would you say it is:
    □ Not hard at all   □ Somewhat hard   □ Very hard

12. Which of these categories is closest to your total annual household income, including income from all family members living with you?
    (This includes all income from jobs, pensions, interest investments, Social Security, or other payments.)
   - □ Less than $20,000
   - □ $20,000 – < $40,000
   - □ $40,000 – <$60,000
   - □ $60,000 – <$80,000
   - □ $80,000 – <$100,000
   - □ $100,000 or more
